# Poroelastic Modeling of Highly Hydrated Collagen Hydrogels: Experimental Results vs. Numerical Simulation With Custom and Commercial Finite Element Solvers

**DOI:** 10.3389/fbioe.2018.00142

**Published:** 2018-10-23

**Authors:** André P. G. Castro, Jiang Yao, Tom Battisti, Damien Lacroix

**Affiliations:** ^1^IDMEC, Instituto Superior Técnico, University of Lisbon, Lisbon, Portugal; ^2^Department of Mechanical Engineering, Insigneo Institute for in silico Medicine, University of Sheffield, Sheffield, United Kingdom; ^3^Dassault Systèmes Simulia Corp., Johnston, RI, United States

**Keywords:** biomechanics, biomaterials, biomedical engineering, soft tissues, collagen, finite element solvers

## Abstract

This study presents a comparison between the performances of two Finite Element (FE) solvers for the modeling of the poroelastic behavior of highly hydrated collagen hydrogels. Characterization of collagen hydrogels has been a widespread challenge since this is one of the most used natural biomaterials for Tissue Engineering (TE) applications. V-Biomech® is a free custom FE solver oriented to soft tissue modeling, while Abaqus® is a general-purpose commercial FE package which is widely used for biomechanics computational modeling. Poroelastic simulations with both solvers were compared to two experimental protocols performed by Busby et al. (2013) and Chandran and Barocas (2004), also using different implementations of the frequently used Neo-Hookean hyperelastic model. The average differences between solvers outputs were under 5% throughout the different tests and hydrogel properties. Thus, differences were small enough to be considered negligible and within the variability found experimentally from one sample to another. This work demonstrates that constitutive modeling of soft tissues, such as collagen hydrogels can be achieved with either V-Biomech or Abaqus standard options (without user-subroutine), which is important for the biomechanics and biomaterials research community. V-Biomech has shown its potential for the validation of biomechanical characterization of soft tissues, while Abaqus' versatility is useful for the modeling and analysis of TE applications where other complex phenomena may also need to be captured.

## Introduction

Since collagen is a natural biomaterial, intrinsically biocompatible and biodegradable, collagen-based hydrogels are widely used in tissue engineering (TE). These collagen hydrogels can be used as a scaffold as they present an advantageous host for cell migration, proliferation and differentiation (Cen et al., 2008; Sharabi et al., 2014). Collagen applications vary from nervous system models to anticancer drugs testing, since collagen is used as a scaffold or as a membrane for electrophysiological protocols (Deponti et al., 2014). Several research groups worked on collagen characterization, which is still a challenge, due to its complexity and wide-range of applications (Roeder et al., 2002; Castro et al., 2016). Collagen fibers are known to be anisotropic and have great influence on cartilaginous tissues and structures, such as the annulus fibrosus of the intervertebral disc, which leads to non-linear modeling approaches (Caner et al., 2007; Marini and Ferguson, 2014; Wismer et al., 2014; Long et al., 2016). For hydrogels, the most accurate modeling approaches include poro-viscoelastic theories considering their multiphasic behavior and time-dependency (Busby et al., 2013; Xu et al., 2013; Castro et al., 2016). Collagen modeling is challenging and has been discussed over the years, namely in what concerns to compressibility, fiber contribution and biphasic behavior, so this work intends to present different alternatives for the biomaterials research community.

In this work, experimental characterization of collagen hydrogels reported by Chandran and Barocas (2004) and Busby et al. (2013) is reproduced with two different Finite Element (FE) solvers, namely the custom poroelastic solver V-Biomech® presented by Castro et al. (Castro et al., 2014, 2016) and commercial FE package Abaqus® 6.13 (Dassault Systèmes Simulia Corp., USA). Other FE solvers, such as FEBio (free) or COMSOL (commercial), could also be considered for poroelastic modeling. As an example, some studies have already compared the behavior of Abaqus and FEBio for contact problems (Meng et al., 2013, 2017; Galbusera et al., 2014), while FEBio developers have also validated their calculations against Abaqus (Maas et al., 2012). V-Biomech has already shown good accuracy for biphasic osmotic swelling problems, also when compared with Abaqus-based models (Castro et al., 2014).

Historically, there has been a discussion on Abaqus ability to model complex strain-dependent poroelastic problems without additional user-defined subroutines (Prendergast et al., 1996; van der Voet, 1997; Wu et al., 1998). Such subroutines are still required for osmotic swelling or fibrillar modeling (Barthelemy et al., 2016; Fallah et al., 2016), but this work intends to evaluate the performance of one open-source poroelastic FE solver (V-Biomech, free to download^1^) and “out of the box” (without using user-defined subroutines) commercially available FE package Abaqus on the already challenging task of modeling the highly non-linear behavior of collagen hydrogels. This also leads to the comparison of different implementations of the Neo Hookean model, in order to evaluate if relevant differences are raised by compressible and incompressible formulations. Ultimately, this work aims to contribute to the definition of a framework for other *in vitro* and *in silico* combined works that make use of related soft biomaterials or hydrogels (Girton et al., 2002; Silva-Correia et al., 2011; Freutel et al., 2014; Chaudhuri et al., 2015).

## Materials and methods

The FE simulations replicated the protocols of the ramp-hold confined compression experiments of Chandran and Barocas (2004) and Busby et al. (2013). The first group used cuboid hydrogel samples of 3 × 3 × 15 mm, with 0.30% of bovine collagen concentration (by weight). The protocol was divided into compression and relaxation stages: (i) 10% compression during 100 s and, (ii) compression hold for 2,000 s. The latter group used cylindrical samples with a radius of 8 mm and a height of 5 mm, and considered hydrogels with 0.20, 0.30, and 0.40% of rat-tail collagen concentration (by weight). The protocol was also divided into compression and relaxation stages: (i) 5% compression during 10 s and, (ii) compression hold for 290 s.

The FE modeling strategy (meshes, boundary conditions and materials) was the same for both V-Biomech and Abaqus, regardless of the intrinsic specifications of each solver. It must also be highlighted that only standard modeling options were chosen, i.e., no alterations were made on V-Biomech for these specific tests (Castro et al., 2014, 2016; Cortez et al., 2017) and no user-defined subroutines were added to Abaqus. The major setup difference is on the graphical interfaces: while Abaqus has the option between its full graphical interface or input/output file generation, V-Biomech simulations are solely defined through dedicated input files for mesh, boundary conditions, material constitutive modeling and output requests. V-Biomech pre- and post-processing operations are preferentially performed on GiD® 12.0.7 (CIMNE, Spain).

**Figure 1:**
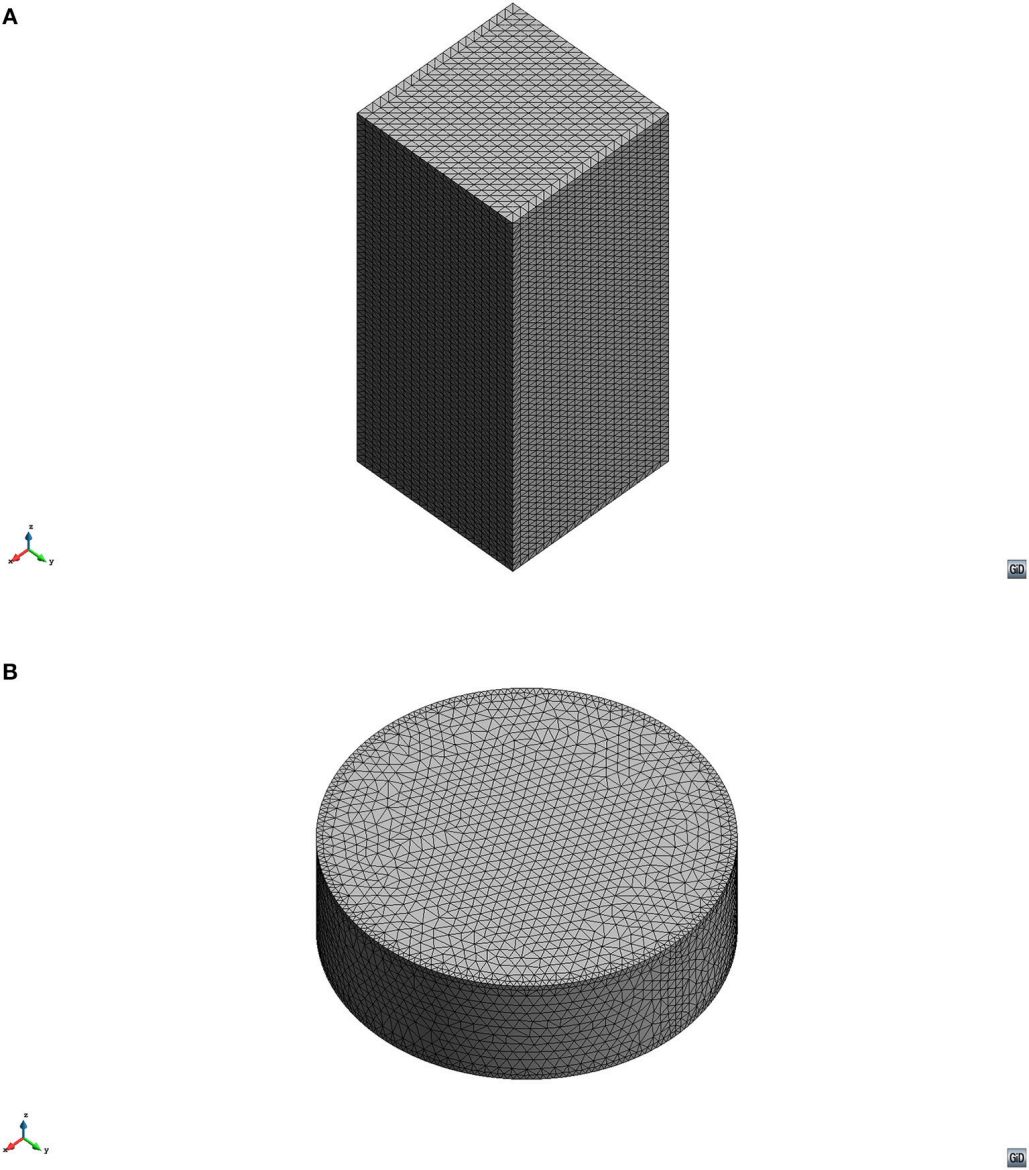
FE models used in this study: **(A)** Cuboidal model to mimic Chandran and Barocas (2004) samples; **(B)** Cylindrical model to mimic Busby et al. (2013) samples.

The FE meshes were generated with GiD and then exported to each solver. Both models (Figure 1) used quadratic 10-node tetrahedral elements: the cuboidal model consisted of 203,401 nodes and 144,000 elements, whereas the cylindrical model consisted of 119,890 nodes and 83,787 elements.

**Figure 2:**
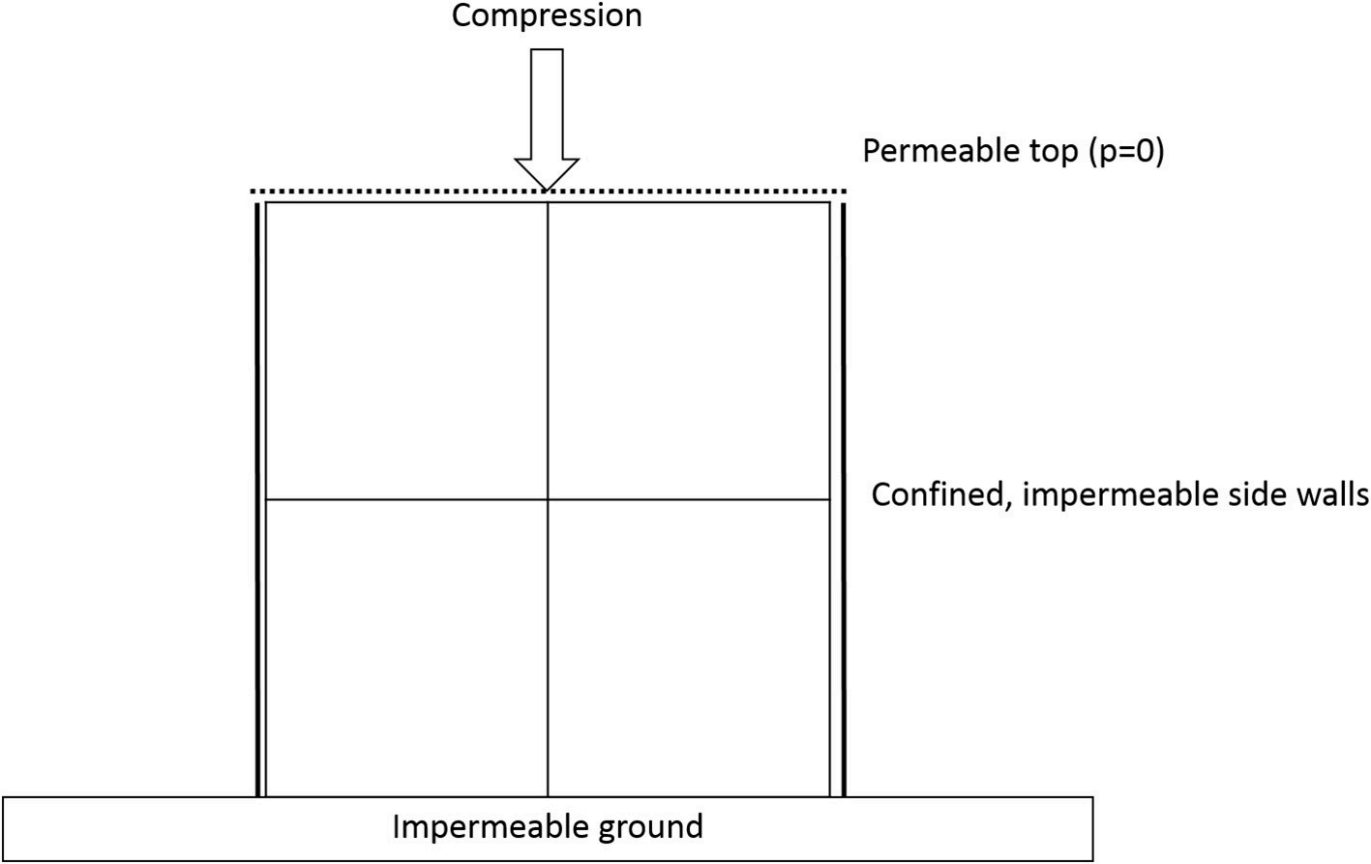
Schematic representation of the boundary conditions applied in this study.

The boundary conditions (Figure 2) for both protocols were implemented as bottom and lateral confinement (X- and Y-axis) with a compression applied at the top (Z-axis). Fluid exudation was allowed through the top by using a null pore pressure condition.

Collagen hydrogels were modeled as hyper-poroelastic (Noailly et al., 2008; Kalyanam et al., 2009; Castro et al., 2016), using the following variations of the Neo-Hookean hyperelastic model:

(1) $${\bar{\text{W}}}_{NH}\left(C\right)=C_{10}\;\left(I_{1}-3\right)$$

(2) $${\text{W}}_{NH}\;\left(C\right)=\frac{G}{2}\left(I_{1}-3\right)-G(\text{ln}J)-\frac{G}{3}{\left(\text{ln}J\right)}^{2}+\frac{K}{2}{\left(\text{ln}J\right)}^{2}$$

(3) $${\bar{W}}_{NH}(C)=C_{10}\;(\bar{I_{1}}-3)+\frac{1}{D_{1}}{\left(J-1\right)}^{2}$$

V-Biomech makes use of the standard incompressible model (Equation 1, ahead referred as NH1) dependent on *C10* (stiffness parameter, related to shear modulus), and also of the compressible model detailed by Bonet and Wood (Bonet and Wood, 1997) (Equation 2, ahead referred as NH2), which depends on shear (*G*) and bulk (*K*) moduli. The model implemented in Abaqus (Equation 3) adds to this the *D*_1_ parameter (compressibility parameter, related to bulk modulus) to define generic compressibility. *C* is the left Cauchy-Green deformation tensor, *J* is the determinant of the deformation gradient tensor, *I*__1__ is the first invariant of *C* and, finally, ${\overset{\text{\_}}{I}}_{{}_{1}}$ is the deviatoric component of the latter. As so, V-Biomech presents a clear distinction between compressible and incompressible modeling strategies, while Abaqus provides a generic Neo-Hookean model by default.

The strain-dependent permeability $\left[k^{*}\left(J\right)\right]$ was considered through van der Voet model (van der Voet, 1997; Castro et al., 2014; Taffetani et al., 2014):

(4) $$K^{*}\left(J\right)=K_{0}^{*}J^{M}$$

$K_{0}^{*}$ is the zero-strain hydraulic permeability and *M* is a dimensionless nonlinear permeability parameter.

Knapp et al. (1997) identified a Poisson's ratio range of 0.2–0.3 for collagen hydrogels, while previous work with dynamic rheology experiments (Castro et al., 2016) suggested that the higher Poisson's ratio values (closer to 0.5) would be a better fit. Since confined compression experiments are more sensitive to the fluid exudation and constitutive properties of the solid components of the hydrogels (Knapp et al., 1997; Laity et al., 2000; Chandran and Barocas, 2004; Kalyanam et al., 2009), the constitutive properties of the collagen hydrogels were based on 0.2 Poisson's ratio value (Busby et al., 2013; Castro et al., 2016). Further details on material modeling can be found in Castro et al. (2016). A summary of the parameters used for the constitutive models is given in Table 1.

**Table 1:** Constitutive properties of the different collagen hydrogels (Busby et al., 2013; Castro et al., 2016).

**Collagen concentration(%)**	$K_{0}^{*}$(m^4^/Ns)	**M**	***G* (kPa)**	***C*_10_ (kPa)**	***K* (kPa)**	***D*_1_ (kPa)**
0.20	1.70 × 10^−10^	1.8	0.3375	0.1688	0.4500	4.444
0.30	1.20 × 10^−10^	2.1	0.3750	0.1875	0.5000	4.000
0.40	0.80 × 10^−10^	3.5	0.4500	0.2250	0.6000	3.333

The numerical output compared was longitudinal effective stress (${\sigma_{e}}_{{}_{zz}^{}}$) plots vs. time at the bottom layer of the samples. It must be highlighted that longitudinal stress ($\sigma_{zz}^{}$) which can be extracted from Abaqus by default does not represent the actual effective stress (σ_*e*_), given to the biphasic configuration of the material. To obtain the effective stress, one needs to output both pore pressure (*p*) and the required stress component, being the effective stress calculated as follows:

(5) $$\sigma_{ezz}=p-\sigma_{zz}$$

Four confined compression simulations were performed with Abaqus: one with the cuboid model (0.30% collagen hydrogel) and three with the cylindrical model (0.20, 0.30, and 0.40% collagen hydrogels). Analogously, eight simulations were performed with V-Biomech, corresponding to the two different Neo-Hookean models available. The emphasis was on evaluating and comparing the performance of the solvers under different testing configurations and material properties.

## Results

**Figure 3:**
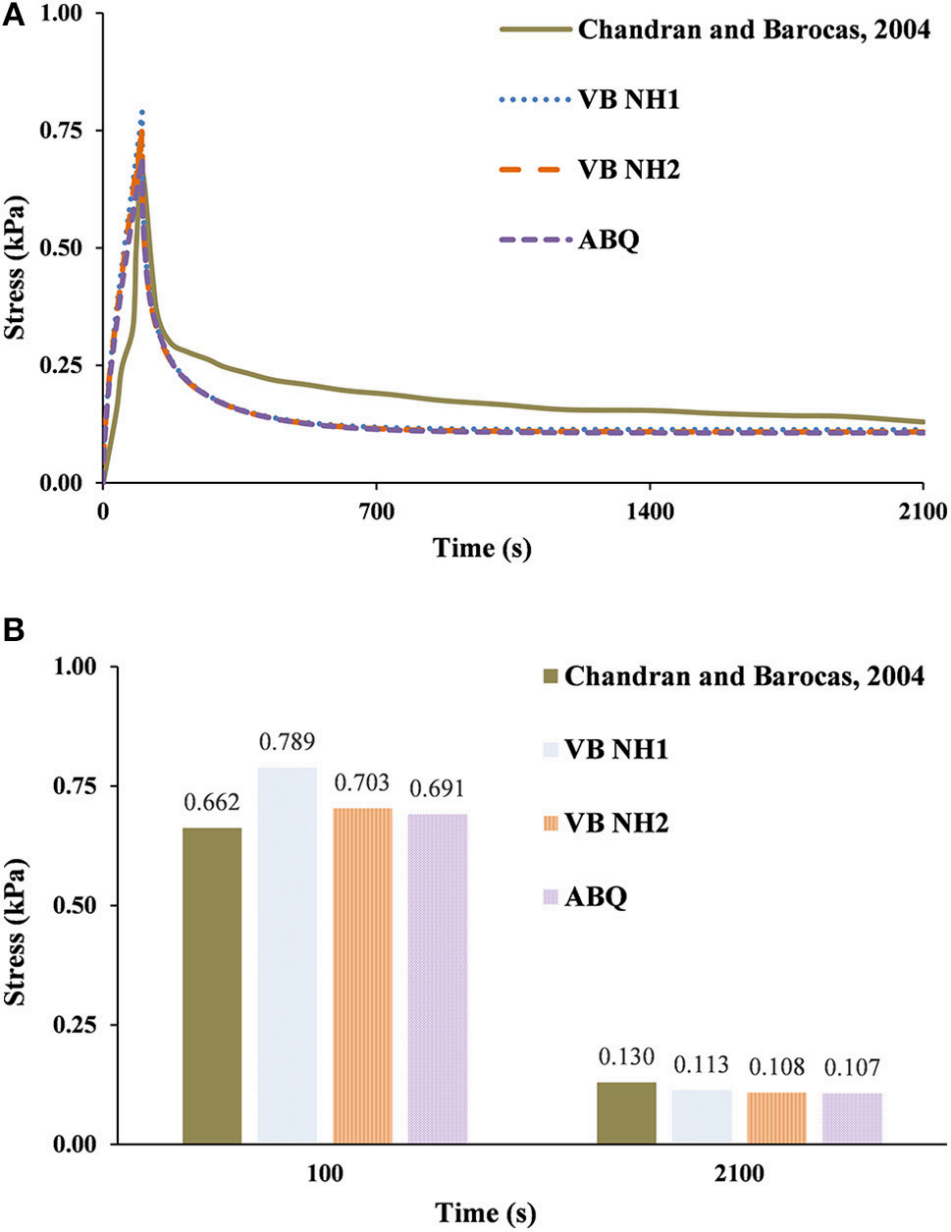
0.30% collagen hydrogel during 2,100 s stress relaxation test. The experimental data of Chandran and Barocas (2004) is here compared with numerical calculations using V-Biomech and Abaqus: **(A)** Stress relaxation over time; **(B)** Peak and end effective stress values.

Figure 3 shows the comparison between the experimental stress curve of Chandran and Barocas (2004) and the numerical calculations with V-Biomech (VB NH1 and VB NH2) and Abaqus (denoted as ABQ). The mechanical behavior of collagen under confined compression is reproduced similarly with both solvers. The experiments showed slower stress relaxation than the numerical models. The peak stress values of the FE calculations are closer to those reported by Chandran and Barocas (2004) than the stress relaxation values. It must be highlighted that no experimental standard deviation was provided. Peak stress values from VB NH1 were on average 19% higher than the experimental results, and on average 13% lower at the end of the test. VB NH2 calculations were 6% higher in peak stress and 16% lower in relaxation stress. Finally, in what concerns to Abaqus, the peak stress value was 4% higher than the experimental results, while the relaxation stress was 18% lower. It is worth noting that the average absolute difference is virtually null between Abaqus and VB NH2 models.

**Figure 4:**
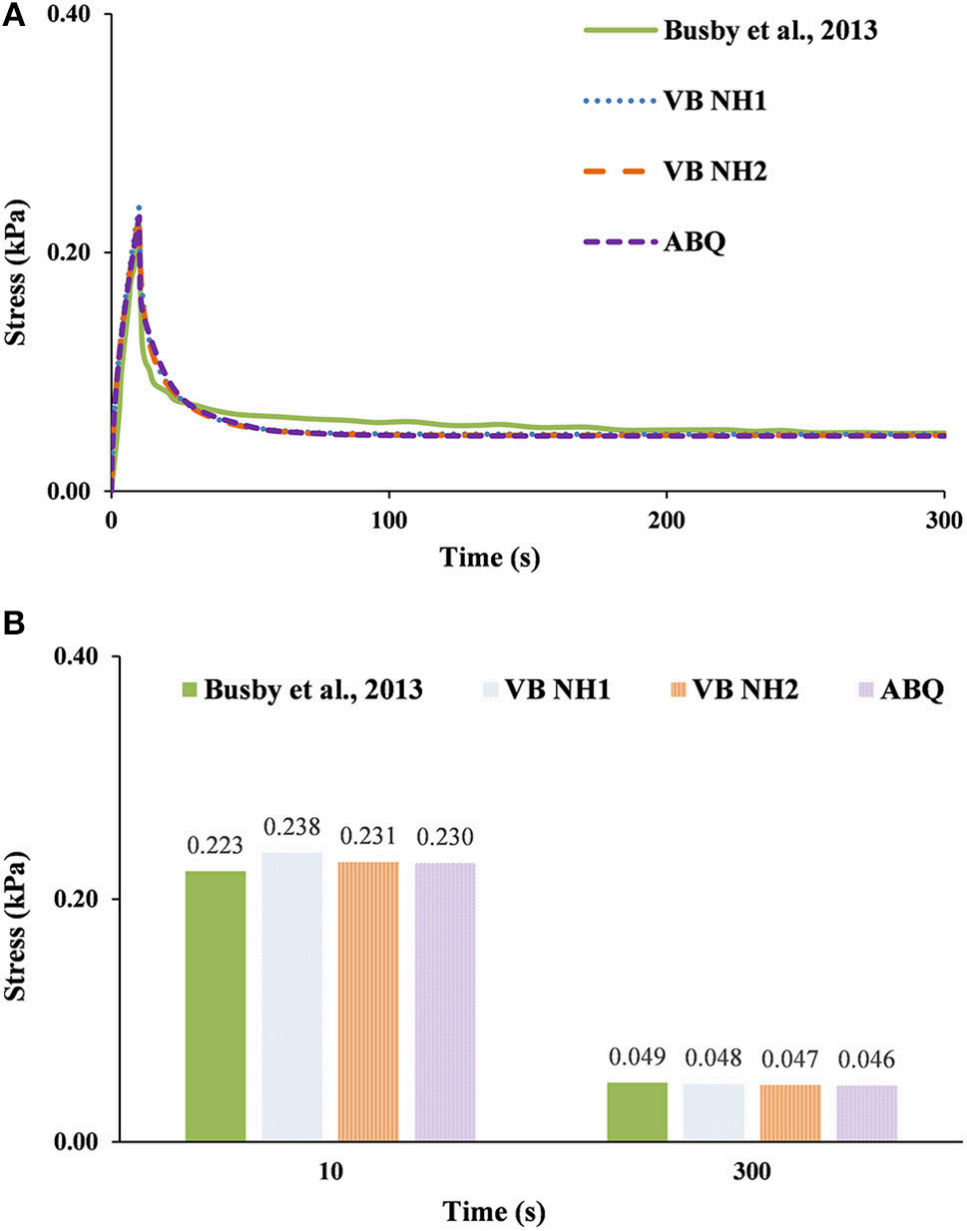
0.20% collagen hydrogel during 300 s stress relaxation test. The experimental data of Busby et al. (2013) is here compared with numerical calculations using V-Biomech, and Abaqus: **(A)** Stress relaxation over time; **(B)** Peak and end effective stress values.

**Figure 5:**
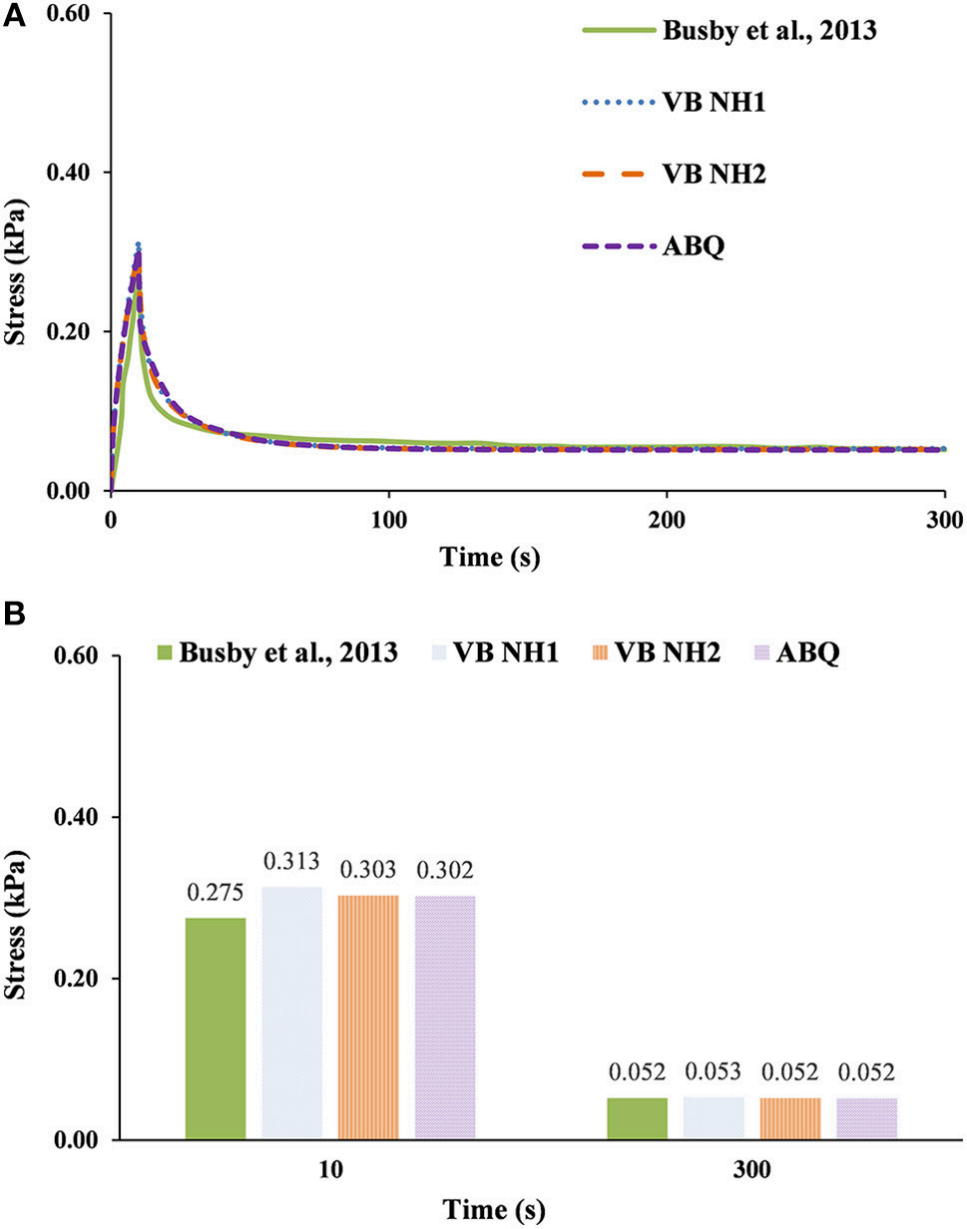
0.30% collagen hydrogel during 300 s stress relaxation test. The experimental data of Busby et al. (2013) is here compared with numerical calculations using V-Biomech, and Abaqus: **(A)** Stress relaxation over time; **(B)** Peak and end effective stress values.

**Figure 6:**
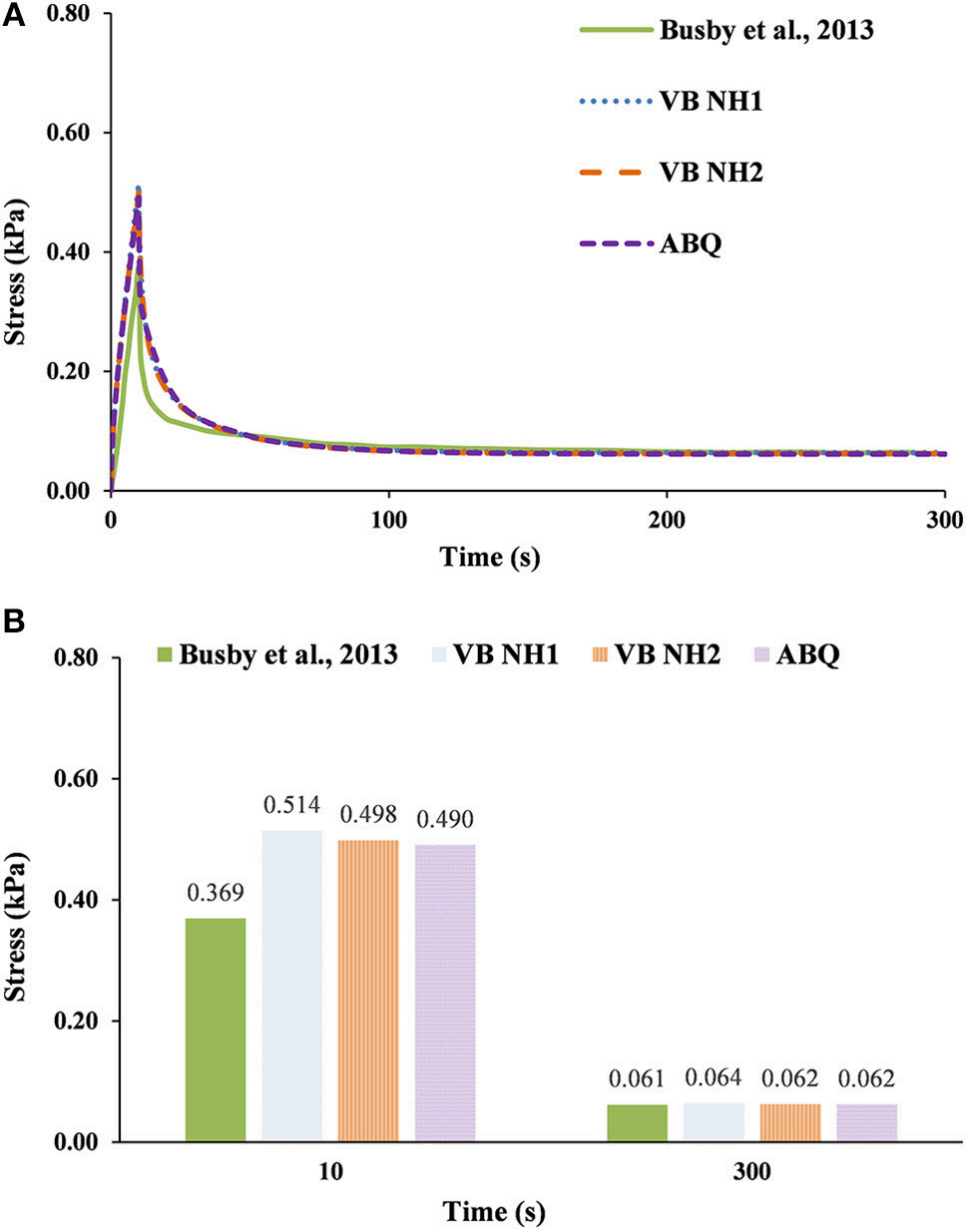
0.40% collagen hydrogel during stress relaxation tests. The experimental data of Busby et al. (2013) is here compared with numerical calculations using V-Biomech, and Abaqus: **(A)** Stress relaxation over time; **(B)** Peak and end effective stress values.

Figures 4–**6** plot the comparison between the evolution of the experimental stress curves of Busby et al. (2013); (knowing that the average standard deviation of the experimental results is ±5%) and the analogous numerical calculations with V-Biomech and Abaqus. Overall, these plots show similar patterns to those previously observed in Figure 3, namely that the numerical simulations predicted a slower stress decrease than what occurred in the experiments (Busby et al., 2013). This is seen across all hydrogel groups and it is also independent of the FE solver chosen.

For the 0.20% group (Figure 4), VB NH2 and ABQ have shown peak stress values 3% higher than the experimental results, while the relaxation stress was 4% lower for VB NH2 and 5% lower for ABQ. For VB NH1, the peak stress value was 7% higher, while the relaxation stress was 2% lower. Hence, differences in this case are inside the ±5% standard deviation of the experiments, being 4% in average for the three models.

For the 0.30% group (Figure 5), VB NH2 and ABQ have shown peak stress values 10% higher than the experimental results, while both relaxation stress values were under 1% of difference to the reference. VB NH1 has shown a peak stress value 14% higher than the experimental results, whereas the relaxation stress was 2% higher. The numerical peak stress values are outside the ±5% standard deviation of the experiments, but the three models are again producing similar results.

Finally, for the 0.40% group (Figure 6), using VB NH1 model, the calculated peak stress value was 39% higher than the experimental results, and the relaxation stress was 4% higher at the end of the test. VB NH2 has shown 35 and 2%, while ABQ has shown 33 and 1%, correspondingly. As so, the average differences ranged from 17% for ABQ to 21% for VB NH1.

## Discussion

This work shows that different modeling options can be equally accurate when one is interested in understanding and replicating collagen hydrogels' behavior, namely in what concerns to solid mechanical model and fiber content evaluation.

The differences compared to the average experimental results in Busby et al. (2013) increased when the collagen concentration was increased, for the three models. VB NH1 has shown 4% average difference for 0.20%, 5% for 0.30%, and 18% for 0.40% (overall average of 11%). For VB NH2, the calculated average differences were 4% for 0.20%, 8% for 0.30%, and 21% for 0.40% (overall average of 9%). Lastly, differences observed with Abaqus were 4% for 0.20%, 5% for 0.30%, and 17% for 0.40% (overall average of 9%). The overall average differences were vastly influenced by the larger offset observed for the 0.40% collagen concentration hydrogel. It is then possible that the conversion from aggregate modulus to Young's modulus [please refer to Busby et al. (2013) and Castro et al. (2016) for further details] may be losing accuracy when the collagen concentration was increased (or for deformations above 10%). Nevertheless, the 2% average difference between the calculations with the three models is lower than the standard deviation of the experiments in Busby et al. (2013) (±5% average across the three collagen hydrogels groups), which most likely means that such differences are not significant for comparing the accuracy of each model against the experiments.

The three models showed similar trends in determining the longitudinal effective stress over time: faster stress relaxation than in Chandran and Barocas (2004), but slower in all the three collagen hydrogels groups investigated in Busby et al. (2013). The experimental conditions are likely to have played in a role in such findings, with particular emphasis for the possibility of friction to occur on the cuboid setup used in Chandran and Barocas (2004), thus impeding the water to flow out in ideal conditions and creating stress accumulation. The comparison with Chandran and Barocas (2004) is then limited by the “ideal” conditions predicted in the numerical models, i.e., friction or interface phenomena were not considered in this work, but could have enhanced the reproduction of the experiments and therefore reduced the calculated differences (11% for Abaqus and VB NH2). It must be highlighted that the intrinsic incompressibility of VB NH1 is the most probable cause for this model to be more distant to the reference (16% average difference) than the other two models, since a lower Poisson's ratio was used. Even though, the overall average differences are not significantly apart from the other two models. No standard deviation information was provided in the work of Chandran and Barocas (2004), but the average absolute difference between VB NH1 and the other models (~5%) may be considered as acceptable and is aligned with the good agreement observed in the comparison with Busby et al. (2013).

## Conclusions

V-Biomech presents the advantage of being a free tool (Castro et al., 2014), which can be modified through its source code if new challenges are presented. The option for different compressible and incompressible models is justified with the potential accuracy increment in soft tissue modeling. On the other side, Abaqus presents the advantage of being able of simulating other complex nonlinear phenomena (such as contact or large deformation) that occurs in advanced TE applications. This solver may also be expanded through user-defined subroutines.

The power of choice over free and commercial FE packages is an advantage for the scientific community interested in numerical modeling and characterization of soft tissues. It is shown here that the standard poroelastic modeling options presented by both solvers allowed for accurate constitutive modeling of collagen hydrogels, which is highly relevant to study other hydrogels, soft tissues and TE applications.

## Author contributions

AC prepared the manuscript, ran the simulations, and compiled the results. JY evaluated the simulations, proposed alternatives, and revised the manuscript. TB discussed the outcomes, and revised the manuscript. DL directed the study, discussed the methods and the outcomes and revised the manuscript.

### Conflict of interest statement

JY and TB would like to disclose that they are employees of Dassault Systèmes Simulia Corporation (USA), responsible for the development of Abaqus. The remaining authors declare that the research was conducted in the absence of any commercial or financial relationships that could be construed as a potential conflict of interest.

## Footnotes

^1^https://github.com/INSIGNEO/VBiomech
